# Relationship between Malaria Incidence and IgG Levels to *Plasmodium falciparum* Merozoite Antigens in Malian Children: Impact of Hemoglobins S and C

**DOI:** 10.1371/journal.pone.0060182

**Published:** 2013-03-28

**Authors:** Kazutoyo Miura, Mahamadou Diakite, Ababacar Diouf, Saibou Doumbia, Drissa Konate, Abdoul S. Keita, Samuel E. Moretz, Gregory Tullo, Hong Zhou, Tatiana M. Lopera-Mesa, Jennifer M. Anderson, Rick M. Fairhurst, Carole A. Long

**Affiliations:** 1 Laboratory of Malaria and Vector Research, National Institute of Allergy and Infectious Diseases, National Institutes of Health, Rockville, Maryland, United States of America; 2 Faculty of Medicine, Pharmacy and Odontostomatology, University of Bamako, Bamako, Mali; Burnet Institute, Australia

## Abstract

Heterozygous hemoglobin (Hb) AS (sickle-cell trait) and HbAC are hypothesized to protect against *Plasmodium falciparum* malaria in part by enhancing naturally-acquired immunity to this disease. To investigate this hypothesis, we compared antibody levels to four merozoite antigens from the *P. falciparum* 3D7 clone (apical membrane antigen 1, AMA1-3D7; merozoite surface protein 1, MSP1-3D7; 175 kDa erythrocyte-binding antigen, EBA175-3D7; and merozoite surface protein 2, MSP2-3D7) in a cohort of 103 HbAA, 73 HbAS and 30 HbAC children aged 3 to 11 years in a malaria-endemic area of Mali. In the 2009 transmission season we found that HbAS, but not HbAC, significantly reduced the risk of malaria compared to HbAA. IgG levels to MSP1 and MSP2 at the start of this transmission season inversely correlated with malaria incidence after adjusting for age and Hb type. However, HbAS children had significantly lower IgG levels to EBA175 and MSP2 compared to HbAA children. On the other hand, HbAC children had similar IgG levels to all four antigens. The parasite growth-inhibitory activity of purified IgG samples did not differ significantly by Hb type. Changes in antigen-specific IgG levels during the 2009 transmission and 2010 dry seasons also did not differ by Hb type, and none of these IgG levels dropped significantly during the dry season. These data suggest that sickle-cell trait does not reduce the risk of malaria by enhancing the acquisition of IgG responses to merozoite antigens.

## Introduction

*Plasmodium falciparum* malaria remains one of the greatest global health problems [1]. The mortality associated with this disease is believed to have evolutionarily selected for hemoglobin (Hb) S (β_6_ Glu to Val) in Africa. This hypothesis is supported by epidemiological studies showing that heterozygosity for ‘adult’ HbA and ‘sickle’ HbS (HbAS, sickle-cell trait) protects children from developing severe, life-threatening malaria syndromes [2]. While some studies [3]–[5] have found that HbAS also protects against uncomplicated *P. falciparum* malaria, this has not been a consistent finding [6], [7]. Several mechanisms are proposed to explain how HbAS confers malaria protection, including: (i) restricted parasite invasion and/or growth in HbAS erythrocytes, especially under conditions of low oxygen tension; (ii) enhanced phagocytosis of parasitized HbAS erythrocytes by macrophages; and (iii) impaired cytoadherence of parasitized HbAS erythrocytes to microvascular endothelial and other host cells [8]. A recent study in mice has suggested that modulation of carbon monoxide levels by HbS may also exert malaria-protective effects [9]. In addition to such innate factors, current studies have indicated that acquired immunity may also contribute to the protective mechanism [3], [10]. Passive transfer studies in African children with *P. falciparum* malaria have verified the importance of immune IgG in the clearance of blood-stage parasitemias [11]. However, the effector mechanisms of this IgG-mediated protection and the impact of Hb variants on protective IgG responses have not been completely elucidated.

The HbC variant is also produced by a single amino acid substitution (β_6_ Glu to Lys). The effects of HbAC on the incidence of uncomplicated and severe *P. falciparum* malaria are less well established than those of HbAS [2]. For example, studies have reported that HbAC confers protection against both uncomplicated and severe malaria [12], against severe malaria only [13], or specifically against the cerebral form of severe malaria [14]. On the other hand, some studies suggest that HbAC confers protection against neither uncomplicated [4], [5] nor severe [15], [16] malaria. Collectively, these epidemiological studies suggest that HbAS and HbAC may exert their malaria-protective effects in part by different mechanisms.

To investigate whether IgG responses to *P. falciparum* merozoite antigens contribute to the malaria-protective effects of HbAS and HbAC, we conducted a prospective sub-cohort study in Mali. The sub-cohort included all HbAS and HbAC children aged 3 to 11 years and a paired group of HbAA children who were matched as closely as possible for age and potential malaria-protective erythrocyte polymorphisms (α-thalassemia, glucose-6-phosphate dehydrogenase (G6PD) deficiency, ABO/Rh blood group antigens). Plasma samples were collected at the start of the transmission season (May 2009), at the end of the transmission season (December 2009), and at the end of the subsequent dry season (May 2010). We measured antigen-specific IgG levels to four merozoite antigens (*P. falciparum* 3D7 clone) and analyzed their relationship to malaria incidence in the 2009 transmission season.

## Methods

### Cohort studies and blood collection

In May 2008, we initiated a 4-year longitudinal cohort study of Malian children (aged 0.5 to 17 years) in three villages (Kenieroba, Fourda, Bozokin) where malaria transmission is intense and seasonal (June to December/January). The details of the study site and cohort have been described elsewhere [17]. All children were typed for erythrocyte polymorphisms (Hb type, α-thalassemia, G6PD deficiency, ABO/Rh blood group antigens), as described [4], [18]. From this cohort, we enrolled 73 HbAS and 30 HbAC children, aged 3 to 11 years, into a sub-cohort study. Children were not enrolled from Bozokin for logistical reasons. For each HbAS or HbAC child, we enrolled a HbAA child who was completely matched for age and matched as closely as possible for erythrocyte polymorphism profile (Table S1). The resulting sub-cohort included 70, 80 and 56 children who were 3–5, 6–8 and 9–11 years old, respectively (Table S2).

At the start of the sub-cohort study (May 2009), we obtained plasma samples and stored them at −80°C until use. The parents or guardians of all children enrolled in the cohort study were activity encouraged to bring their child to our study clinic if they developed symptoms of malaria. Uncomplicated malaria was defined as axillary temperature >37.5°C (or history of fever in the past 48 hours) with or without other symptoms of malaria (e.g., headache, malaise), any density of asexual *P. falciparum* parasites on microscopic examination of a thick blood film, and no other etiologies of febrile illness discernible on clinical examination. Severe malaria was defined by WHO criteria [19]. Plasma samples were also collected at the end of the transmission season (December 2009, n = 191), and at the end of the subsequent dry season (May 2010, n = 180).

The cohort and sub-cohort studies were approved by the Ethics Committee of the Faculty of Medicine, Pharmacy and Odontostomatology, University of Bamako, and the Institutional Review Board (IRB) of the National Institute of Allergy and Infectious Diseases (NIAID). Adult parents or guardians of children gave written informed consent if they could read French. If they could not read French, oral consent was obtained by the study physician and a finger print obtained from the consenting adult. A literate third-party individual witnessed both informed consent processes and signed the informed consent document. The Ethics Committee at University of Bamako and the IRB of NIAID approved the informed consent procedure. The study is registered with Clinicaltrials.gov, number NCT00669084.

### Measurement of antigen-specific IgG levels by ELISA

The standardized method for performing the ELISA was described previously [20]. In brief, flat-bottom 96-well ELISA plates were coated with 100 ng test protein per well, and plasma samples tested in triplicate at 1∶500–1∶50,000 dilutions. The absorbance at 405 nm was read using a microplate reader, and the absorbance of individual test samples was converted into ELISA units using a standard curve generated by serially diluting the standard in the same plate. The ELISA unit value of a standard was assigned as the reciprocal of the dilution giving an O.D. _405_ = 1 in a standardized assay. Apical membrane antigen 1 (AMA1)-3D7 [21] and merozoite surface protein 1 (MSP1, 42 kDa)-3D7 [22] were kindly provided by Dr. David Narum (NIAID/NIH), erythrocyte-binding antigen 175kDa-3D7(EBA175-3D7) region II [23] protein by Drs. Annie Mo and Lee Hall (NIAID/NIH), and merozoite surface protein 2 (MSP2)-3D7 [24] by Dr. Robin Anders (La Trobe University, Melbourne, Australia). The minimal detection level of IgG in this study was 44 ELISA units; all responses below this level were assigned a value of 22 ELISA units.

To measure anti-tetanus toxoid IgG titers and determine IgG subclass, we randomly selected plasma samples from eight pairs of matched HbAA and HbAS children in each age group (3–5, 6–8 and 9–11 years) and tested them (n = 48) at a 1∶500 dilution. Tetanus toxoid was purchased from List Biological Laboratories, Inc. (Campbell, CA).

### Measurement of parasite growth inhibitory activity of IgG by GIA

The growth inhibition assay (GIA) was conducted with protein G-purified total IgGs and the standardized method was described previously [25]. In brief, test IgGs (at a final concentration of 10 mg/ml per well), synchronized *P. falciparum* parasites (3D7 clone) and culture medium were added to 96-well tissue culture plates in triplicate and cultured for ∼40 h. Relative parasitemias were quantitated by biochemical determination of parasite lactate dehydrogenase. Percent inhibition of test IgG was calculated as 100 - [(A_650_ of test IgG - A_650_ of normal RBCs)/(A_650_ of infected RBCs without any IgG - A_650_ of normal RBCs) ×100].

### Statistical analysis

Categorical variables were compared using a chi-squared test. Times to first malaria episode were compared by a log-rank test. Continuous variables between two groups were compared by a Mann-Whitney test, and those between three groups were compared by a Kruskal-Wallis test (if significant, Dunn's multiple comparison test was then used). For paired comparisons, a Wilcoxon signed-rank test was used. Spearman's correlation test was used to assess correlation between two data sets. We used a nominal logistic fit of data for malaria experience (i.e., whether or not malaria was experienced), a Cox proportional hazards regression for time to first malaria episode and a longitudinal analysis for number of malaria episodes. For these multivariate regression analyses, age (as a continuous variable), Hb type (HbAS or non-HbAS) and log_10_-transformed ELISA units were used as variables. To determine whether there was a significant interaction between age and Hb type on the time to first malaria episode, a Cox regression analysis was performed with age, Hb type and the cross-product of these two variables. Data were analyzed using Prism 5 (GraphPad Software, Inc., San Diego, CA) or JMP8 (SAS Institute, Inc., Cary, NC). p-values <0.05 were considered significant.

## Results

### HbAS is associated with reduced malaria risk in Malian children

**Figure 1:**
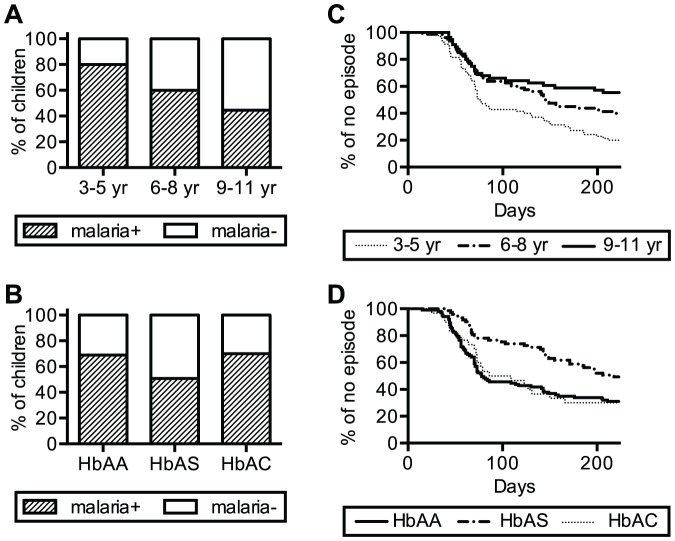
Increasing age and HbAS significantly protect Malian children against malaria. (A & B) The proportion of children who experienced at least one episode of malaria (malaria+) in the 2009 transmission season is shown. The children are categorized by age (A) or Hb type (B). (C & D) Survival analyses of the time to first malaria episode. Kaplan-Meier plots are shown for children grouped by age (C) or Hb type (D). Increasing age and HbAS were significantly associated with reduced malaria risk as measured by whether or not malaria was experienced (Figure 1A, p<0.0001; Figure 1B, p = 0.0203; chi-squared tests), or time to first malaria episode (Figure 1C, p = 0.0002; Figure 1D, p = 0.0013; by log-rank tests).

In May 2009, we identified all HbAS (n = 73) and HbAC (n = 30) children in a cohort of Malian children from two villages. For each HbAS or HbAC child, we enrolled a HbAA child who was completely matched for age. The erythrocyte polymorphism profile (i.e., ABO/Rh blood group antigen phenotypes, G6PD*A- and α-globin^−3.7kb^ genotypes) was matched as closely as possible (Table S1). There were no significant differences in the demographic characteristics and erythrocyte polymorphisms between the groups of HbAA, HbAS and HbAC children (Table S2). Only four children (2 HbAA, 1 HbAS, 1 HbAC) had asymptomatic *P. falciparum* parasitemia (50–200/ µl whole blood) at enrollment. A total of 206 children were followed passively for malaria episodes throughout the 2009 transmission season (June 1, 2009 to January 31, 2010). Increasing age and HbAS were significantly associated with reduced malaria risk, as measured by: (i) whether or not malaria was experienced (Figure 1A, p<0.0001; Figure 1B, p = 0.0203; chi-squared tests), and (ii) time to first malaria episode (Figure 1C, p = 0.0002; Figure 1D, p = 0.0013; log-rank tests). HbAA and HbAC children did not differ significantly in these measures of malaria risk.

### HbAS is associated with lower merozoite antigen-specific IgG levels at the start of the transmission season

**Figure 2:**
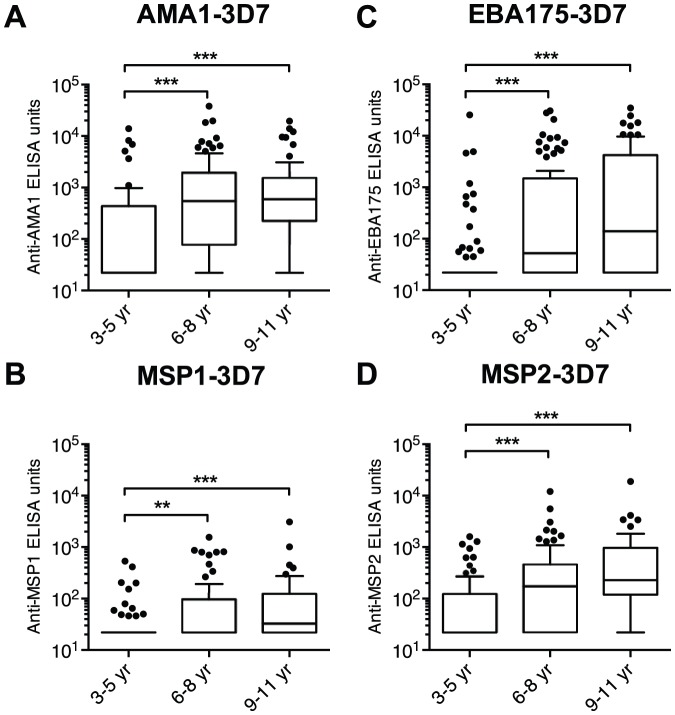
Merozoite antigen-specific IgG levels increase with age. Box and whisker (Tukey) plots of IgG levels in each age group are shown. The levels of IgG to AMA1-3D7 (A), MSP1-3D7 (B), EBA175-3D7 (C) and MSP2-3D7 (D) were quantified. All responses below the limit of detection (44 ELISA units) were assigned a value of 22 ELISA units. IgG levels between the three age groups were compared using a Kruskal-Wallis test followed by Dunn's multiple comparison test (**, p<0.01; ***, p<0.001).

**Figure 3:**
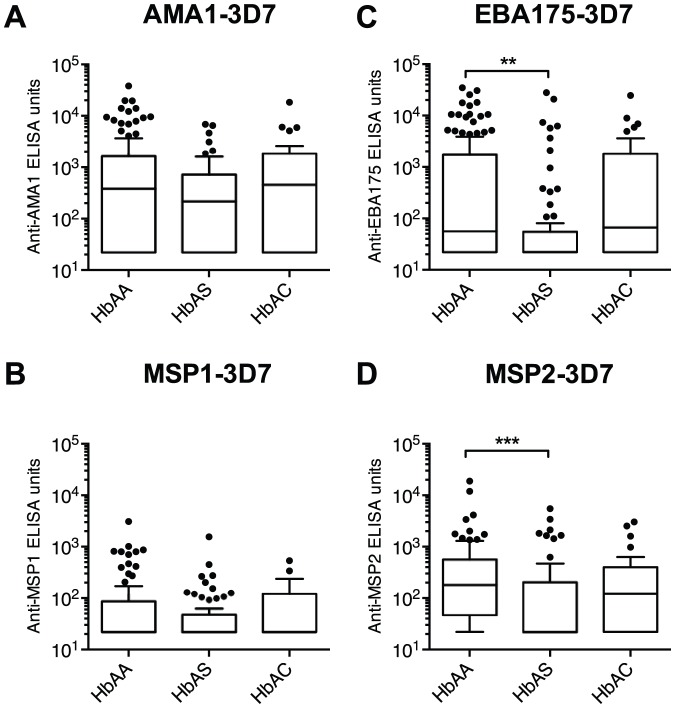
Merozoite antigen-specific IgG levels are lower in HbAS children. Box and whisker (Tukey) plots of IgG levels in each Hb type group are shown. The levels of IgG to AMA1-3D7 (A), MSP1-3D7 (B), EBA175-3D7 (C) and MSP2-3D7 (D) were quantified. All responses below the limit of detection (44 ELISA units) were assigned a value of 22 ELISA units. IgG levels between the three Hb type groups were compared using a Kruskal-Wallis test followed by Dunn's multiple comparison test (**, p<0.01; ***, p<0.001).

To explore whether enhanced humoral immunity contributed to the malaria-protective effects of sickle-cell trait, we quantified plasma IgG levels to four merozoite antigens in HbAA, HbAS and HbAC children just prior to the transmission season (May 2009). In the entire sub-cohort of children, we found strong correlations (r_s_ = 0.49–0.67, p<0.0001) between all combinations of antigen-specific IgG levels (Figure S1). The older two age groups showed significantly higher antigen-specific IgG levels than the youngest age group, while there was no significant difference between the older two groups (Figure 2). Compared to HbAA children, HbAS children had significantly lower levels of EBA175- and MSP2-specific IgG and tended to have lower levels of AMA1- and MSP1-specific IgG (Figure 3). In contrast, none of these IgG levels differed between HbAC and HbAA children (Figure 3). After adjusting for age and Hb type, other erythrocyte polymorphisms (i.e., ABO and Rh phenotypes, G6PD*A- and α^−3.7kb^ genotypes) were not associated with antigen-specific IgG levels (data not shown).

To determine whether HbAS children had lower IgG levels specifically for merozoite antigens, we quantified tetanus toxoid-specific IgG titers in eight randomly selected pairs of matched HbAA and HbAS children in each age group (3–5, 6–8 and 9–11 years). We found no differences in these IgG levels between HbAA and HbAS children (data not shown). In these paired HbAA and HbAS plasma samples, we also found no differences in IgG subclass distributions. Regardless of Hb type, IgG_1_ was dominant for AMA1, MSP1 and EBA175, IgG_3_ was dominant for MSP2, and IgG_2_ and IgG_4_ were detected at near-background levels for all four merozoite antigens.

Antibody functionality was evaluated by a growth inhibition assay (GIA) using protein G-purified IgGs. We found significant correlations between antigen-specific IgG levels and growth-inhibitory activities (r_s_ = 0.27–0.49; p<0.0001 for AMA1, EBA175 and MSP2; p = 0.0002 for MSP1). The median growth-inhibitory activity of purified IgGs was 23.0% (IQR, 9.0–50.0) in HbAS children, 23.0% (IQR, 16.0–38.8) in HbAC, and 36.0% (IQR, 16.0–54.0) in HbAA. The difference among the three Hb types did not reach significance (p = 0.054 by Kruskal-Wallis test).

### Merozoite antigen-specific IgG levels inversely correlate with malaria risk

To explore whether IgG responses to merozoite antigens contributed to malaria protection in our sub-cohort of Malian children, we evaluated correlations between both ELISA and GIA data and malaria risk. In the multivariate regression analysis, children were categorized as either HbAS or non-HbAS (i.e., HbAA or HbAC) since malaria risk did not differ between HbAC and HbAA children (Figure 1). After adjusting for age and Hb type, only MSP1- and MSP2-specific IgG levels inversely correlated with measures of malaria risk (i.e., whether a malaria episode was experienced, time to first malaria episode, or number of malaria episodes) when all of four IgG levels were analyzed together (Table 1). The growth-inhibitory activity of IgG did not correlate with any measure of malaria risk after adjusting for age and Hb type (data not shown).

**Table 1:** Results of multivariate regression analysis adjusted for age and Hb type^a^.

	Whether malaria was experienced			Time to first malaria episode			Number of malaria episodes		
	OR^b^	(95% CI)	p	HR^c^	(95% CI)	p	IRR^d^	(95% CI)	p
AMA1-3D7	1.11	(0.66–1.92)	0.689	1.10	(0.82–1.47)	0.534	1.07	(0.87–1.33)	0.513
MSP1-3D7	0.41	(0.17–0.94)	0.034	0.45	(0.23–0.83)	0.010	0.59	(3.7–0.89)	0.012
EBA175-3D7	0.85	(0.53–1.37)	0.511	1.02	(0.76–1.37)	0.874	1.05	(0.85–1.30)	0.650
MSP2-3D7	0.49	(0.24–0.97)	0.039	0.59	(0.38–0.90)	0.013	0.63	(0.46–0.85)	0.002

a For these multivariate regression analyses, age (as a continuous variable), Hb type (HbAS or non-HbAS) and log_10_-transformed ELISA units were used as variables.

b OR, odds ratio

c HR, hazard ratio

d IRR, incident rate ratio

### Changes in merozoite antigen-specific IgG levels from one transmission season to the next

**Figure 4:**
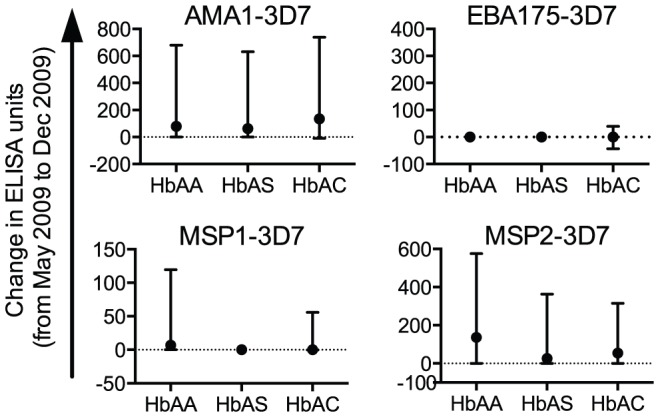
Increases in merozoite antigen-specific IgG levels during a transmission season do not differ by Hb type. For each child, the change in IgG level from May to December 2009 was calculated for each antigen (A, AMA1-3D7; B, MSP1-3D7; C, EBA175-3D7; D, MSP2-3D7). All responses below the limit of detection (44 ELISA units) were assigned a value of 22 ELISA units. Median and interquartile range are shown. Positive values represent increases in IgG levels at the end of the transmission season. Changes in IgG levels were not significantly different among the three Hb types for any antigen (p>0.05 by Kruskal-Wallis tests).

At beginning of the 2009 transmission season, HbAS children had lower antigen-specific IgG levels than HbAA children. To explain this finding, we first hypothesized that these IgG levels increase less in HbAS children than in HbAA children over the course of a transmission season. To test this hypothesis, we collected paired plasma samples from HbAA (n = 97, 94.2% of those collected in May 2009), HbAS (n = 68, 93.2%) and HbAC (n = 26, 86.7%) children in December 2009. At this time, 18 HbAA (18.6%), 12 HbAS (17.6%) and 5 HbAC (19.2%) children had asymptomatic *P. falciparum* parasitemia (25–1875/ µl whole blood). The IgG levels to all four merozoite antigens were higher in December 2009 than in May 2009 (p<0.001, Wilcoxon matched-pairs signed rank tests). The increases in these IgG levels did not differ significantly by Hb type (Figure 4) and did not correlate with number of malaria episodes in the 2009 transmission season (data not shown).

**Figure 5:**
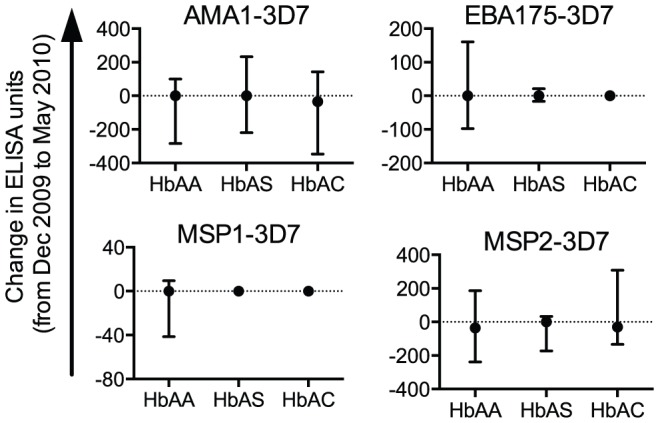
Merozoite antigen-specific IgG levels do not decay during a dry season, regardless of Hb type. For each child, the change in IgG level from December 2009 to May 2010 was calculated for each antigen (A, AMA1-3D7; B, MSP1-3D7; C, EBA175-3D7; D, MSP2-3D7). All responses below the limit of detection (44 ELISA units) were assigned a value of 22 ELISA units. Median and interquartile range are shown. Positive values represent increases in IgG levels at the end of the dry season. Changes in IgG levels were not significantly different among the three Hb types for any antigen (p>0.05 by Kruskal-Wallis tests).

To further investigate the basis for lower antigen-specific IgG levels in HbAS children in May 2009, we hypothesized that these IgG levels decay faster in HbAS children than in HbAA children over the course of a dry season. To test this possibility, we collected paired plasma samples from HbAA (n = 89, 91.7% of those collected in May 2009), HbAS (n = 64, 94.1%) and HbAC (n = 27, 86.7%) children in May 2010. At this time, none of these children had microscopically detectable *P. falciparum* parasitemia. The IgG levels to all four merozoite antigens were not significantly different between December 2009 and May 2010 (p>0.05). These changes in antigen-specific IgG levels did not differ significantly by Hb type (Figure 5). Taken together, the data suggest that Malian children annually increase their levels of anti-merozoite IgGs during transmission season and maintain these antibody levels throughout the 5-months dry season.

**Figure 6:**
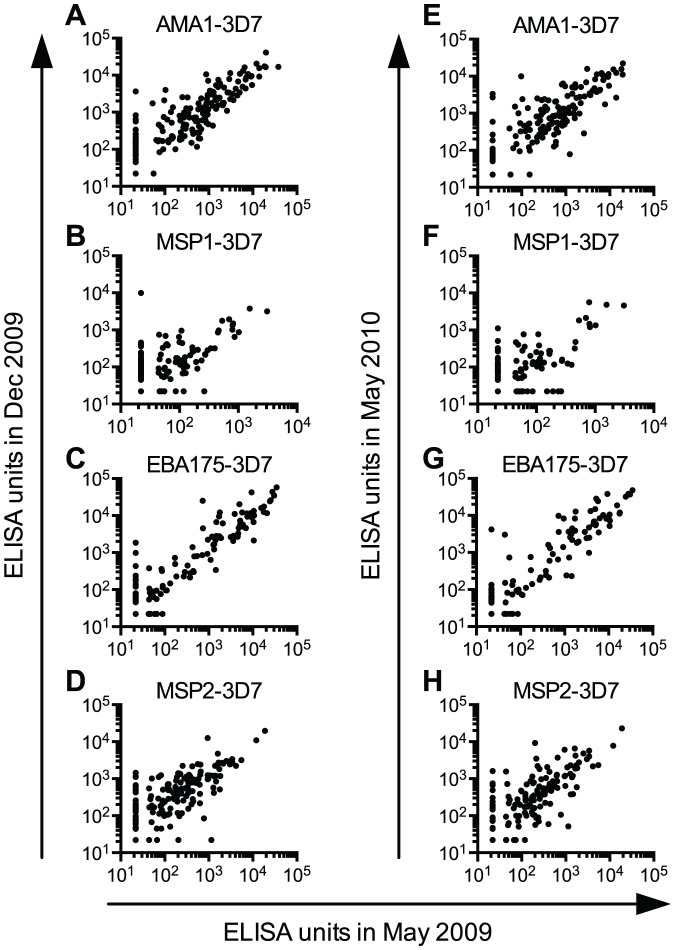
Significant correlations between merozoite antigen-specific IgG levels at three time points. IgG levels in May 2009 are plotted against IgG levels in December 2009 (A–D) and May 2010 (E–H). Each dot represents data from a single child. Plasma IgG levels to four merozoite antigens (A and E, AMA1-3D7; B and F, MSP1-3D7; C and G, EBA175-3D7; D and H, MSP2-3D7) are correlated. All responses below the limit of detection (44 ELISA units) were assigned a value of 22 ELISA units. All correlations shown are significant (p<0.001 by Spearman rank tests).

In the entire sub-cohort, we also compared antigen-specific IgG levels at three different time points. There were strong positive correlations (r_s_ = 0.59–0.89, p<0.0001) between these IgG levels in May and December 2009 (Figures 6A-D) and in May 2009 and May 2010 (Figures 6E-H). These data suggest that children with relatively low levels of anti-merozoite IgGs in the study population at May 2009 remained low (and children with high level remained high) throughout one year of the study period, while there were significant increases during transmission season.

## Discussion

Previous studies investigating the impact of HbAS and HbAC on the development of *P. falciparum*-specific IgG responses have produced conflicting results. Some studies have shown that HbAS children have higher [26], [27], similar [28]–[30], or lower [31], [32] IgG titers to *P. falciparum* antigens than HbAA children. Conflicting data have also been reported for HbAS children within individual studies, dependent on antigens used [6], IgG subclasses measured [33], [34], and epidemiological setting studied [35]. Other studies have shown that HbAC children have similar [30] or enhanced [35] IgG responses to *P. falciparum* antigens compared to HbAA children. These suggest that the effect of β-globin mutations on humoral responses to merozoite antigens may be altered by other host factors (e.g., children or adults, etc.), environmental factors (e.g., malaria endemicity, etc.) and/or antigens used for assay. Using highly standardized ELISA and GIA assays, we have showed that HbAS children had lower IgG levels to EBA175 and MSP2, and a reduced risk of *P. falciparum* malaria, compared to HbAA children. HbAS children also had lower IgG levels to AMA1 and MSP1, but these did not differ significantly from those in HbAA children. These data suggest that HbAS does not confer protection against malaria in our study population by enhancing IgG responses to merozoite antigens.

While we measured IgG levels to only four merozoite antigens, the strong correlations between these IgG levels within individuals suggest that HbAA and HbAS children differ in their exposure to *P. falciparum* antigens generally. Cholera *et al*. reported that levels of *P. falciparum* erythrocyte membrane protein 1 (PfEMP1) are lower on the surface of HbAS compared to HbAA erythrocytes [36]. Given reports of abnormal PfEMP1/knob display on the surface of parasitized HbAS and HbAC erythrocytes [2], [36], [37], we are also evaluating antibody responses to the surface of parasitized erythrocytes in a separate study (A. Zeituni *et al.*, submitted). If HbAS causes quantitative or qualitative changes in merozoite antigens exposed to the immune system during erythrocyte invasion, these changes may explain the lower IgG levels to merozoite antigens in HbAS children. However, to our knowledge, studies have not yet compared antigen levels on the surface of merozoites released from parasitized HbAS, HbAC and HbAA erythrocytes. Alternatively, some studies have reported that the parasite density in HbAS children was lower than that in HbAA children [5], [10], [34]. The lower parasite burden in HbAS children might reduce the amount of antigens presented to the immune system, resulting in lower IgG titers. However, one study showed no difference in parasite density between HbAS and HbAA children [6], and in another study a parasite density difference was observed only in children of given ages [31]. Further investigation is needed to explain the lower antigen-specific IgG levels in HbAS children in our study population.

Observations that IgG titers to a single antigen can correlate significantly with clinical protection in one study, but not in another [38], suggest that the antimalarial effect of IgG responses are altered by host and parasite factors. To best evaluate relationships between IgG levels and malaria risk, it is important to account for the malaria-protective effects of Hb variants simultaneously in the same study population. However, few longitudinal studies have collected immunological and clinical data from both HbAS and HbAA children in the same epidemiological setting [6], [31]. Since these studies did not report correlations between immunological responses and clinical outcomes, the contribution of specific immune responses measured in these studies to antimalarial immunity in the specific study populations is difficult to assess. For example, anti-MSP1 titer measured in a study may not contribute to the clinical immunity in the particular study population. In this study, we measured both immunological and clinical data, then show that HbAS children had significantly lower MSP2 titers than HbAA children, while the high level of anti-MSP2 antibody significantly reduced the malaria risk after adjusting for age and Hb type in the same study population.

Previous studies have suggested a contribution of acquired immunity to protection in HbAS children, evidenced by the observation that the level of protection against malaria increases with age to a greater extent in HbAS than in HbAA children [3], [10]. In the present study, we tested whether increasing age reduced the malaria risk to the same degree both in HbAS and HbAA children (data not shown). When we performed a Cox regression analysis (with the time to first malaria episode as a readout) using age, Hb type and the cross-product of these two variables, the cross-product had no significant impact (p = 0.345). Neither of the previous studies, which involved larger numbers (1,054 [3] or 601 [10]) of children and longer periods (∼2 years [3] or 18 months [10]) of observation, showed statistically significant interactions between age and Hb type. Identifying the putative components of acquired immunity that contribute to malaria protection by HbAS may thus require even larger datasets. Further studies are required to discover whether IgG responses to additional antigens and other components of acquired immunity mediate some of the malaria protection conferred by HbAS.

In this study, HbAC and HbAA children did not differ in malaria risk or IgG levels to four merozoite antigens. There were relatively fewer HbAC children (n = 30) than HbAS children (n = 73) in this study site, which might reduce the power to detect the differences between HbAC and HbAA children. However, we did not see any trend (e.g., fewer malaria episode, lower IgG levels) in HbAC children. In addition, since the effects of HbAC on malaria risk [4], [5], [12]–[16] and antibody levels [30], [35] vary widely between studies, our findings were not surprising. While some studies have found significant correlations between GIA data and malaria risk [39], [40], our study did not. This apparent discrepancy may be explained in part by varying incidence of malaria, prevalence of Hb variants, and levels of antimalarial immunity in the different study populations. Such discrepancies among different studies emphasize the importance of evaluating immunological and clinical measurements at the same time in the same study population.

We found no evidence that antigen-specific IgG levels are lower in HbAS children because they increase less during the transmission season or decay more rapidly in the dry season than in HbAA and HbAC children. These IgG levels increased in all children during the 2009 transmission season, but the fold-increase in IgG levels did not correlate with the number of malaria episodes the children experienced. This finding suggests that increases in antigen-specific IgG levels during a transmission season do not necessarily result from the development of a malaria episode. Instead, asymptomatic infections may play an important role in developing *P. falciparum*-specific IgG responses.

Once they increased, antigen-specific IgG levels did not decay over the 5-month dry season. This finding is surprising since others have reported that *P. falciparum*-specific antibodies are short-lived (e.g., half-life of a few weeks) [41]–[43] compared to anti-viral antibodies with half-lives of 10 to >200 years [44]. However, caution should be exercised in this comparison since the two phases of antibody kinetics (the acute clearance phase within ∼1 month after antigen exposure, and the stable phase [45]) can have completely different half-lives for the same immunogen. Other studies have found the half-lives of *P. falciparum*-specific antibodies to be relatively longer, especially in adults [46], [47]: one study [46] showed no significant reduction in titers against MSP1 antigens during a 6-month dry season, and the other study [47] estimated that the half-lives of anti-AMA1/MSP1 antibodies were 5–10 years. The strong correlations in IgG levels measured at two different time points (Figure 6) suggest that we measured most IgG levels in their stable phase.

The mechanisms of long-term antibody maintenance by memory B cells and long-lived plasma cells are poorly understood, especially in humans. In part, this is because the predominant site of antibody production is the bone marrow where up to 90% of long-lived plasma cells reside [45]. Indeed, the numbers (or presence) of antigen-specific memory B cells, which have been differentiated from peripheral blood mononuclear cells *ex vivo*, do not always correlate with the level (or presence) of antigen-specific antibodies [44], [47], [48]. It is difficult to know whether asymptomatic parasitemia during the dry season continuously stimulated the immune system to sustain the IgG levels we measured. Our two measurements of asymptomatic parasitemia (2% in May 2009, 0% in May 2010) suggest that microscopically-detected *P. falciparum* infection was very uncommon in our study population during the dry season. However, the prevalence of sub-patent parasitemia could have been substantially higher if we used a more sensitive method of detection, such as polymerase chain reaction. Further study is required to uncover the mechanism of sustained antigen-specific IgG levels during the dry season.

In summary, HbAS and HbAC differentially associated with malaria risk and merozoite antigen-specific IgG levels in Malian children. HbAS children had lower IgG levels to four merozoite antigens, suggesting that these IgG responses – two of which correlated with reduced malaria incidence – do not mediate the malaria-protective effects of HbAS in 3- to 11-year-old children. The development and decay of IgG levels through the transmission and dry seasons did not differ between HbAS and HbAA children, suggesting that the differences in their IgG levels may have been established before 3 years of age.

## Supporting Information

Figure S1 **Significant correlations between merozoite antigen-specific IgG levels.** IgG levels in individual plasma samples against two different merozoite antigens are compared in each panel. All responses below the limit of detection (44 ELISA units) were assigned a value of 22 ELISA units. All correlations shown are significant (p<0.001, Spearman rank test).
(EPS)

Table S1 **Detail of Malian children pairs.**
(XLSX)

Table S2 **Demographic characteristics and erythrocyte polymorphisms of Malian children, stratified by hemoglobin type.** ^a^ p-values were calculated using Chi-square tests. ^b^ α-thalassemia genotypes for two HbAS children were not determined.
(DOCX)
