# Supplementary material for: Relationship between Malaria Incidence and IgG Levels to Plasmodium falciparum Merozoite Antigens in Malian Children: Impact of Hemoglobins S and C
Source: PLoS One. 2013 Mar 28;8(3):e60182. doi: 10.1371/journal.pone.0060182 (PMC3610890; doi:10.1371/journal.pone.0060182)
Supplement: Table S2 — Demographic characteristics and erythrocyte polymorphisms of Malian children, stratified by hemoglobin type. a p-values were calculated using Chi-square tests. b α-thalassemia genotypes for two HbAS children were not determined. (DOCX) [file pone.0060182.s003.docx]

|  | |  | **HbAA**  **(n=103)** |  | **HbAS**  **(n=73)** |  | **HbAC**  **(n=30)** | **p-value**^a^ |
| --- | --- | --- | --- | --- | --- | --- | --- | --- |
| **Age** | | |  |  |  |  |  | 0.996 |
|  | 3-5 years | | 35 |  | 25 |  | 10 |  |
|  | 6-8 years | | 40 |  | 29 |  | 11 |  |
|  | 9-11 years | | 28 |  | 19 |  | 9 |  |
| **Sex ratio (M/F)** | | | 51/52 |  | 40/33 |  | 14/16 | 0.692 |
| **Village** | | |  |  |  |  |  | 0.451 |
|  | | Kenieroba | 93 |  | 66 |  | 29 |  |
|  | | Fourda | 10 |  | 7 |  | 1 |  |
| **ABO phenotype** | | |  |  |  |  |  | 0.415 |
|  | | A | 36 |  | 24 |  | 11 |  |
|  | | AB | 7 |  | 6 |  | 0 |  |
|  | | B | 20 |  | 16 |  | 4 |  |
|  | | O | 40 |  | 27 |  | 15 |  |
| **Rh phenotype** | | |  |  |  |  |  | 0.563 |
|  | | Positive | 93 |  | 69 |  | 28 |  |
|  | | Negative | 10 |  | 4 |  | 2 |  |
| ***G6PD**A- genotype** | | |  |  |  |  |  | 0.556 |
|  | | Wildtype | 92 |  | 64 |  | 23 |  |
|  | | Heterozygote | 8 |  | 6 |  | 5 |  |
|  | | Hemizygote | 3 |  | 3 |  | 2 |  |
| **α^-3.7kb^ genotype**^b^ | | |  |  |  |  |  | 0.335 |
|  | | Wildtype | 77 |  | 53 |  | 22 |  |
|  | | Heterozygote | 25 |  | 18 |  | 7 |  |
|  | | Homozygote | 1 |  | 0 |  | 1 |  |
